# Case Report: A Case of Late-Onset Combined Methylmalonic Acidemia and Hyperhomocysteinemia Induced by a Vegetarian Diet

**DOI:** 10.3389/fped.2022.896177

**Published:** 2022-07-12

**Authors:** Bei Xu, Lihong Zhang, Qiang Chen, Yajuan Wang, Yahong Peng, Hui Tang

**Affiliations:** ^1^Department of Pediatrics, Baoding No. 1 Central Hospital, Baoding, China; ^2^Department of Emergency, Baoding No. 1 Central Hospital, Baoding, China

**Keywords:** methylmalonic academia, homocysteinemia, metabolic disease, vegetarian diet, pediatrics, China

## Abstract

Methylmalonic acidemia is a rare autosomal recessive metabolic disease. However, because of the atypical clinical symptoms, the type of late-onset methylmalonic academia is often misdiagnosed. Especially when the blood vitamin B12 and folic acid levels are normal, it is not easy to think of this disease. Herein we report a 9-year-old girl who developed normally on a relatively balanced diet before 7 years of age. However, she presented with fatigue and attention deficit when she followed a vegetarian diet. Laboratory examination showed moderate macrocytic anemia, high levels of homocysteine, high level of propionylcarnitine/acetylcarnitine, urinary methylmalonic acid and methyl citrate. Gene mutation analysis showed c.609G > A and c.80A > G compound heterozygous mutations in the *MMACHC* gene, supported late-onset combined methylmalonic academia with homocysteinemia. Then treatment performed with add meat to the diet, vitamin B12, folic acid betaine and L-carnitine supplement. One week later, the child's clinical symptoms and the laboratory examinations were significantly improved.

## Introduction

Methylmalonic acidemia (MMA) is a rare autosomal recessive metabolic disease caused by methylmalonyl-CoA mutase or cobalamin (cbl, also known as vitamin B12) deficiency. In China, cbl C deficiency is the most common type of combined MMA and homocysteinemia, which is caused by congenital defects in cbl metabolism (1, 2). The main manifestations include the increase in urinary methylmalonic acid and blood homocysteine (3). Most patients exhibit an early onset, before 1 year old. These patients are characterized by acute diseases in infancy, such as dysplasia, acute nervous system degeneration, intellectual disability, retinopathy, multiple organ system dysfunction and hematological abnormalities (4, 5). However, there are few reports on combined MMA with homocysteinemia of late-onset cblC (onset after 4 years old), and it is easily misdiagnosed (6).

Here, we report a case of a 9-year girl who presented fatigue and inattention induced by a vegetarian diet. Examination revealed moderate macrocytic anemia, high levels of homocysteine, high level of propionylcarnitine(C3)/acetylcarnitine(C2), urinary methylmalonic acid and methyl citrate. Compound heterozygous mutations of c.609G > A and c.80A > G were found in the *MMACHC* gene. The child was diagnosed with the type of late-onset combined MMA with homocysteinemia and was administered timely treatment to delay disease progression.

## Case Description

A 9-year-old girl presented to our hospital with a 2-year history of fatigue and attention deficit disorder. In the last 3 months, she realized that the above symptoms were aggravated, and she experienced nightmares and became timid. She had shown no obvious abnormal symptoms before that and denied any history of prior disease.

Her previous and personal history was as follows: she was the first child in her family. She had normal birth weight, head circumference, height, and developmental history. She was a mediocre pupil in grade 4 at school. She was not picky about food. Before the age of 7, she had meals in kindergarten 3 times a day. After that, she went to primary school and had meals at home with her grandmother, who was mainly vegetarian. She followed her grandmother's plant-based diet.

The girl's physical examination revealed a normal body development. She had an ear temperature of 37°C, a pulse of 90 beats per minute, and a blood pressure of 90/60 mm Hg. She had clear consciousness but a slow reaction. Her palpebral conjunctiva and lips were pale and appeared mild to moderate anemia. No edema in her eyelids and lower limbs. Cardiopulmonary and abdominal examinations were normal. The muscle strength of her limbs was normal, but the muscle tone of her lower limbs was minimal. Bilateral knee tendon reflex decreased. Her sensations of pain and heat were normal. The joint position sense of the limbs was normal. Her bilateral plantar reflex was normal. Upper limb rotation, finger-to-nose tests and heel-to-shin and Romberg tests were normal.

After admission, we did some tests for her. Routine blood examination showed moderate macrocytic anemia. Hb levels were 84 g/L, red blood cell (RBC) levels were 1.88 × 10^12^/L (normal range, 3.8–5.1 × 10^12^/L), mean corpuscular volume (MCV) was 128.7 fL (normal range, 80–100 fL), red blood cell distribution width (RDW-SD) was 75.50 fL (normal range, 37–50 fL), hematocrit (HCT) was 24.20% (normal range, 35–45%), mean hemoglobin (MCH) was 44.7 pg (normal range, 27–34 pg), platelet (PLT) levels were 425.00×10^9^/L (normal range, 100–300 × 10^9^/L), the reticulocyte percentage was 1.75% (normal range, 0.5–1.5%). Vitamin B12 levels were 389 pg/mL (normal range, 200–1,000 pg/mL), folic acid levels were 22.6 ng/ml (normal range, 5.21–20 nq/ml), and homocysteine increased to 159 umol/L (normal range, 0–15 umol/L). Routine urine analysis showed the following: 36.1 RBC/UL, urine occult blood 2+, urine protein 3+, and negative urine bilirubin. The urinary red blood cell morphology suggested glomerular hematuria. The 24-h urinary protein level was 269.1 mg/24 h (normal range, 28–141 mg/24 h). She had normal urine output, blood creatinine of 68.9 umol/L (normal range, 41.0–73.0 umol/L) and urea was 9.85 mmol/L (normal range, 2.60–7.5 mmol/L), β2-microglobulin 3.02 mg/L (1–3.00 mg/L). Routine fecal and occult blood tests were negative. The levels of blood cholesterol, triglyceride, albumin and hemobilirubin were normal. The coagulation functions were normal. Serum anti-erythrocytic antibodies, anti-IgG, anti-C3, anti-IgG+C3 and free antibody tests were all negative. Ferritin (104.5 ng/mL) and transferrin (1.92 mg/dL) were normal or nearly normal. The patient had no acidemia, and the pH (7.43), HCO${}_{3^{-}}$ (22.0 mmol/L), and BE (-1.5 mmol/L) levels were within the normal range. The anion gap (16.6 mmol/L) was slightly increased. No abnormality was found on magnetic resonance imaging scan of head. No abnormalities in cardiac structure or urinary system were found by color doppler ultrasound.

Then, we obtained the consent of the patient's parents and performed the measure of plasma amino acid and acylcarnitine by tandem mass spectrometry, and the urine concentrations of organic acids by gas chromatography mass spectrometry. The C3/C2 ratio in plasma was increased to 0.43 (normal range, 0.02–0.20). The urinary methylmalonic acid and methylcitrate levels were increased to 92.7 mg/g creatinine (normal range, 0.0–4.0 mg/g creatinine) and 1.8 mg/g creatinine (normal range, 0.0–0.7 mg/g creatinine), respectively.

**Figure 1:**
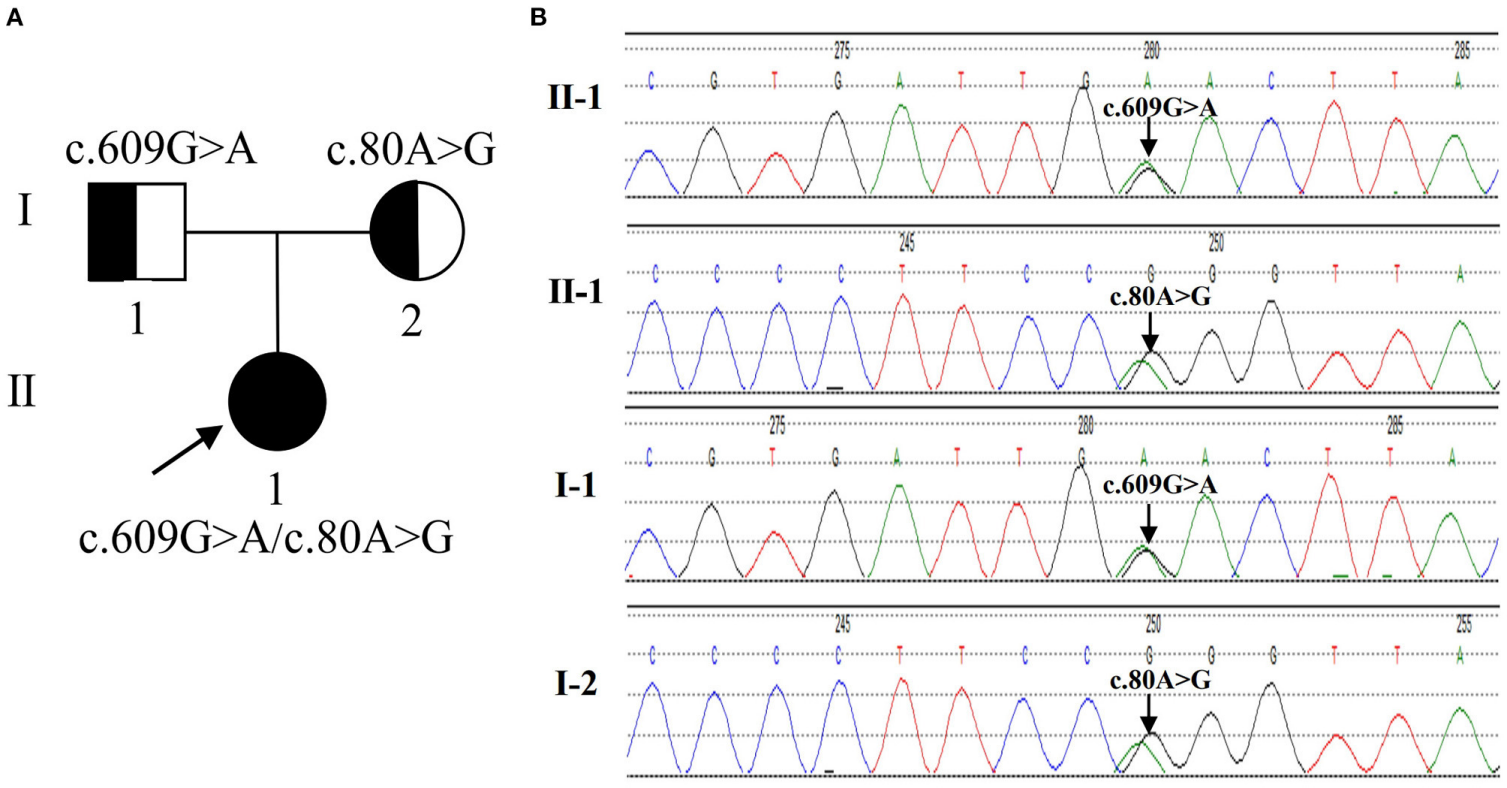
The genetic characteristics of the family with the *MMACHC* mutation. **(A)** The pedigree of the family. The proband is a compound heterozygous mutation in the *MMACHC* gene. Both of their parents are carriers. **(B)** Sequencegrams. *MMACHC* c.609G>A and c.80A>G were identified in the DNA of the patient (II-1). The mutation of *MMACHC* c.609G>A was found in DNA samples derived from his father (I-1). The mutation of *MMACHC* c.80A>G was found in DNA samples derived from his Mather (I-2).

The clinical manifestations and laboratory examination of the patient supported the diagnosis of late-onset combind MMA with homocysteinemia, macrocytic anemia and renal damage. Then, we used next-generation sequencing for gene mutation analysis, revealing c.609G > A (p.W203X) and c.80A > G (p.Q27R) compound heterozygous mutations in the *MMACHC* gene of the proband. Both mutations are pathogenic mutations, Her parents with normal clinical phenotype were c.609G > A and c.80A > G mutation carriers respectively, so the mutation inherited from her parents (Figure 1). The patient is classified as the cblC subtype.

Once the diagnosis was confirmed, the corresponding treatment was performed: the addition of meat to the diet, intramuscular injection of 1 mg/d vitamin B12 for 3 days and then adjusted to 0.5 mg intramuscular injection three times a week, and oral supplementation of 5 mg/d folic acid, betaine 3 g/d and 1 g/d L-carnitine. During the treatment above, her blood pressure and vital signs remained stable without any other new symptoms. One week later, anemia significantly improved: red blood cells increased to 2.2 × 10^12^/L, Hb increased to 90.0 g/L, MCV decreased to 117.0 fL, and the percentage of reticulocytes increased to 6.99%. Plasma homocysteine level decreased to 49.6 μmol/L. The urine protein and occult blood decreased to 1+, the blood urea decreased to 4.6 mmol/L. In addition, the girl's spirit improved, and the reaction was more flexible than before. Her appetite increased, and her weight increased by 0.5 kg in a week.

## Discussion

MMA is a rare autosomal recessive metabolic disorder of methylmalonyl-CoA mutase or its coenzyme cbl that leads to abnormal accumulations of methylmalonic acid, propionic acid, methyl citrate, and other metabolites, causing nerve, liver, kidney, bone marrow, and other organ damage (7–9). Two main forms of the disease have been identified, including isolated MMA and combined MMA (10), which is accompanied by homocysteine. In Europe, America and Japan, isolated MMA is the main type. However, in China, combined MMA with homocysteinemia is the main type, accounting for 70% of all patients (4, 11). Depending on the age of disease onset, MMA patients are divided into the early-onset type (during the first year after birth) and late-onset type (after the age of 4) (12).

CblC is the most common type of combined MMA and homocysteinemia, which is caused by decreased function in vitamin B12 due to mutations in the *MMACHC* gene. The estimated incidence of cblC in Europe and the United States ranges from 1:46,000 to 1:200,000 (13, 14), and it has been deduced from 1:3220 to 1:21,488 (8, 15, 16) in China. Approximately 90% of reported patients with cblC disease present with the severe, infantile, early-onset form of the disease (12, 17, 18). However, late-onset cblC disease is rare, and a patient with atypical clinical symptoms is easily misdiagnosed (6, 19). The clinical symptoms of this child appeared late with large cell anemia accompanied by renal damage. After admission, vitamin B12 and folic acid levels were within the normal range, which could be easily misdiagnosed as renal anemia or digestive system disease. Subsequently, we performed the analysis of plasma amino acids, C3 levels and urine concentrations of organic acids. The results showed that plasma C3, C3/C2, and urine methylmalonic acid levels were increased. Due to the presence of high homocysteine levels, she was clinically diagnosed with combined MMA and homocysteinemia (20). Mutation analysis is more reliable evidence for MMA diagnosis. The child was more than 4 years old at the age of onset; thus, this disease was considered to be a late-onset subtype of combined MMA and homocysteinemia (cblC type) precipitated by vegetarianism. Excessive accumulation of MMA in the blood can cause mitochondrial dysfunction, neuronal apoptosis, oxidative stress, resulting in neurodevelopmental disorders (21). High level of homocysteine may damage endothelial cells and stimulate pro-inflammatory signaling pathways (18), which contribute to the child renal dysfunction and not good academic performance. The clinical symptoms of the child improved significantly after treatment, including the renal functions which improved swiftly and not requiring the need for renal biopsy. In addition, there was no hypertension or abnormality in coagulation factors.

It is worth noting that the vitamin B12 blood levels in these children are within the normal range compared to those in normal individuals. Although vitamin B12 concentrations were normal in our patient, they should be measured to exclude nutritional deficiency disorders of the absorption of vitamin B12 (7). Vitamin B12 is not synthesized in the human body. It is naturally found in animal food products, including meat, poultry, (shell) fish, eggs, milk, and other dairy products (22). Vitamin B12 is generally not present in plant foods. A diet rich in plant-based foods and with fewer animal source foods will increase the risk of vitamin B12 deficiency, especially for people who need more vitamin B12. Diet-associated vitamin B12 deficiency, which can lead to large cell anemia and nervous system damage, occurs only in people who have been on a strict vegetarian diet for a long time without extra supplement (23).

The grandmother of this child was not a strict vegetarian, so she did not show clinical symptoms caused by vitamin B12 deficiency. However, due to the existence of *MMACHC* gene mutations, she demanded for a high dose of vitamin B12 and sufficient calories to maintain methylmalonic acid metabolism and satisfied the need of the growth and development in the body (24, 25). Therefore, the child developed normally on a relatively balanced diet before 7 years of age. However, she presented with non-specific clinical symptoms later when she changed her usual diet. The reason can be relatively insufficient of vitamin B12 intake and also the overall reduction in her calories that led to her ‘catabolic' state leading to production of higher than usual amounts of MMA in her body, and trigger a series of clinical symptoms.

The treatment principles of combined MMA and homocysteinemia are to reduce the generation of metabolic poisons and/or to accelerate their clearance (26). Intramuscular cbl is more effective in reducing homocysteine and methylmalonic acid levels compared with oral administration, and hydroxycobalamin is more effective than methyl or cyanocobalamin for the patients of cblC (27), so we chose intramuscular hydroxycobalamin. Given that L-carnitine promotes the excretion of propionyl- and methylmalonylcarnitine, the tolerance to nutrients causing propionate production such as odd-chain fatty acids should increase. Oral folic acid and betaine reduce blood homocysteine levels (20). In this case, the patient's anemia rapidly improved. Follow-up methylmalonic acid and homocysteine levels were significantly reduced after intramuscular injections of cbl, confirming a good therapeutic effect.

The prognosis of children with combined MMA and homocysteinemia mainly depends on the type of disease, onset and clinical compliance (3). In general, patients who respond to vitamin B12 have a better prognosis, whereas patients with late-onset have a slower clinical progression (28). After 1 week of the above treatment, her blood C3/C2, urine methylmalonic acid and homocysteine levels were significantly reduced, and her clinical symptoms were significantly improved. These results indicate that vitamin B12 treatment was effective in this child. This finding suggests a good prognosis in the near term, but the long-term efficacy still needs to be further observed.

In conclusion, clinicians should pay attention to the possibility of combined MMA when older children have mental symptoms, large cell anemia, high levels of homocysteine, but normal folic acid and vitamin B12 levels, which may also accompanied by other organ function impairment. The girl we report had both mental symptoms and renal damage. The active improvement of blood amino acid and acylcarnitine levels was conducive to timely and correct diagnosis, and early treatment improved and avoided complications as much as possible. Improved gene analysis provides important evidence for clear genotyping and accurate genetic counseling.

## Data Availability Statement

The datasets for this article are not publicly available due to concerns regarding participant/patient anonymity. Requests to access the datasets should be directed to the corresponding author.

## Ethics Statement

Written informed consent was obtained from the participant's legal guardian/next of kin for the publication of any potentially identifiable images or data included in this article.

## Author Contributions

BX and LZ were main contributor in writing the manuscript. QC and YW collected the patient data. YP analyzed the patient data. HT revised the manuscript. All authors read and approved the final manuscript.

## Funding

This work was funded by the Science and Technology Project of Baoding, China (No. 17ZF141). The funding bodies had no role in the design of the study, the collection, analysis, or interpretation of the data, or writing the manuscript.

## Conflict of Interest

The authors declare that the research was conducted in the absence of any commercial or financial relationships that could be construed as a potential conflict of interest.

## Publisher's Note

All claims expressed in this article are solely those of the authors and do not necessarily represent those of their affiliated organizations, or those of the publisher, the editors and the reviewers. Any product that may be evaluated in this article, or claim that may be made by its manufacturer, is not guaranteed or endorsed by the publisher.

## References

[1] Liu MY Yang YL Chang YC Chiang SH Lin SP Han LS. Mutation spectrum of MMACHC in Chinese patients with combined methylmalonic aciduria and homocystinuria. J Hum Genet. (2010) 55:621–6. 10.1038/jhg.2010.81 20631720

[2] Liu Y Liu YP Zhang Y Song JQ Zheng H Dong H. Heterogeneous phenotypes, genotypes, treatment and prevention of 1 003 patients with methylmalonic acidemia in the mainland of China. Zhonghua Er Ke Za Zhi. (2018) 56:414–20. 10.3760/cma.j.issn.0578-1310.2018.06.003 29886603

[3] Hu S Mei S Liu N Kong X. Molecular genetic characterization of cblC defects in 126 pedigrees and prenatal genetic diagnosis of pedigrees with combined methylmalonic aciduria and homocystinuria. BMC Med Genet. (2018) 19:154. 10.1186/s12881-018-0666-x 30157807 PMC6116561

[4] Morel CF Lerner-Ellis JP Rosenblatt DS. Combined methylmalonic aciduria and homocystinuria (cblC): phenotype-genotype correlations and ethnic-specific observations. Mol Genet Metab. (2006) 88:315–21. 10.1016/j.ymgme.2006.04.001 16714133

[5] Wang X Sun W Yang Y Jia J Li C. A clinical and gene analysis of late-onset combined methylmalonic aciduria and homocystinuria, cblC type, in China. J Neurol Sci. (2012) 318:155–9. 10.1016/j.jns.2012.04.012 22560872

[6] Harding CO Pillers DA Steiner RD Bottiglieri T Rosenblatt DS Debley J. Potential for misdiagnosis due to lack of metabolic derangement in combined methylmalonic aciduria/hyperhomocysteinemia (cblC) in the neonate. J Perinatol. (2003) 23:384–6. 10.1038/sj.jp.7210955 12847533

[7] Carrillo-Carrasco N Venditti CP. Combined methylmalonic acidemia and homocystinuria, cblC type II complications, pathophysiology, and outcomes. J Inherit Metab Dis. (2012) 35:103–14. 10.1007/s10545-011-9365-x 21748408 PMC3529128

[8] Han B Cao Z Tian L Zou H Yang L Zhu W. Clinical presentation, gene analysis and outcomes in young patients with early-treated combined methylmalonic acidemia and homocysteinemia (cblC type) in Shandong province, China. Brain Dev. (2016) 38:491–7. 10.1016/j.braindev.2015.10.016 26563984

[9] Zhou X Cui Y. and Han J. Methylmalonic acidemia: Current status and research priorities. Intractable Rare Dis Res. (2018) 7:73–8. 10.5582/irdr.2018.01026 29862147 PMC5982627

[10] Carrillo-Carrasco N Chandler RJ Venditti CP. Combined methylmalonic acidemia and homocystinuria, cblC type. I clinical presentations, diagnosis and management. J Inherit Metab Dis. (2012) 35:91–102. 10.1007/s10545-011-9364-y 21748409 PMC4219318

[11] Shibata N Hasegawa Y Yamada K Kobayashi H Purevsuren J Yang Y. Diversity in the incidence and spectrum of organic acidemias, fatty acid oxidation disorders, and amino acid disorders in Asian countries: selective screening vs. expanded newborn screening. Mol Genet Metab Rep. (2018) 16:5–10. 10.1016/j.ymgmr.2018.05.003 29946514 PMC6014585

[12] Fischer S Huemer M Baumgartner M Deodato F Ballhausen D Boneh A. Clinical presentation and outcome in a series of 88 patients with the cblC defect. J Inherit Metab Dis. (2014) 37:831–40. 10.1007/s10545-014-9687-6 24599607

[13] Cusmano-Ozog K Lorey F Levine S Martin M Nicholas E Packman S. Cobalamin C disease and expanded newborn screening: the California experience. J Investig Med. (2007) 55:S90. 10.1097/00042871-200701010-00090

[14] Weisfeld-Adams JD Morrissey MA Kirmse BM Salveson BR Wasserstein MP McGuire PJ. Newborn screening and early biochemical follow-up in combined methylmalonic aciduria and homocystinuria, cblC type, and utility of methionine as a secondary screening analyte. Mol Genet Metab. (2010) 99:116–23. 10.1016/j.ymgme.2009.09.008 19836982 PMC2914534

[15] Zhou W Li H Wang C Wang X Gu M. Newborn screening for methylmalonic acidemia in a Chinese population: molecular genetic confirmation and genotype phenotype correlations. Front Genet. (2019) 9:726. 10.3389/fgene.2018.00726 30728829 PMC6351470

[16] Guo K Zhou X Chen X Wu Y Liu C Kong Q. Expanded newborn screening for inborn errors of metabolism and genetic characteristics in a Chinese population. Front Genet. (2018) 9:122. 10.3389/fgene.2018.00122 29731766 PMC5920142

[17] Huemer M Diodato D Martinelli D Olivieri G Blom H Gleich F. Phenotype, treatment practice and outcome in the cobalamin-dependent remethylation disorders and MTHFR deficiency: data from the E-HOD registry. J Inherit Metab Dis. (2019) 42:333–52. 10.1002/jimd.12041 30773687

[18] Wang F Han L Yang Y Gu X Ye J Qiu W. Clinical, biochemical, and molecular analysis of combined methylmalonic acidemia and hyperhomocysteinemia (cblC type) in China. J Inherit Metab Dis. (2010) 33(Suppl. 3):S435–442. 10.1007/s10545-010-9217-0 20924684

[19] Jiang YZ Shi Y Shi Y Gan LX Kong YY Zhu ZJ. Methylmalonic and propionic acidemia among hospitalized pediatric patients: a nationwide report. Orphanet J Rare Dis. (2019) 14:292. 10.1186/s13023-019-1268-1 31842933 PMC6915987

[20] Baumgartner MR Hörster F Dionisi-Vici C Haliloglu G Karall D Chapman KA. Proposed guidelines for the diagnosis and management of methylmalonic and propionic acidemia. Orphanet J Rare Dis. (2014) 9:130. 10.1186/s13023-014-0130-8 25205257 PMC4180313

[21] Richard E Jorge-Finnigan A Garcia-Villoria J Merinero B Desviat LR Gort L. Genetic and cellular studies of oxidative stress in methylmalonic aciduria (MMA) cobalamin deficiency type C (cblC) with homocystinuria (MMACHC). Hum Mutat. (2009) 30:1558–66. 10.1002/humu.21107 19760748

[22] Stabler SP. Clinical practice. Vitamin B12 deficiency. N Engl J Med. (2013) 368:149–60. 10.1056/NEJMcp1113996 23301732

[23] Wolffenbuttel BHR Wouters HJCM Heiner-Fokkema MR van der Klauw MM. The many faces of cobalamin (Vitamin B12) deficiency. Mayo Clin Proc Innov Qual Outcomes. (2019) 3:200–14. 10.1016/j.mayocpiqo.2019.03.002 31193945 PMC6543499

[24] Gherasim C Ruetz M Li Z Hudolin S Banerjee R. Pathogenic mutations differentially affect the catalytic activities of the human B12-processing chaperone CblC and increase futile redox cycling. J Biol Chem. (2015) 290:11393–402. 10.1074/jbc.M115.637132 25809485 PMC4416844

[25] Wang H Li L Qin LL Song Y Vidal-Alaball J Liu TH. Oral vitamin B12 versus intramuscular vitamin B12 for vitamin B12 deficiency. Cochrane Database Syst Rev. (2018) 3:CD004655. 10.1002/14651858.CD004655.pub3 29543316 PMC6494183

[26] Huemer M Diodato D Schwahn B Schiff M Bandeira A Benoist JF. Guidelines for diagnosis and management of the cobalamin-related remethylation disorders cblC, cblD, cblE, cblF, cblG, cblJ and MTHFR deficiency. J Inherit Metab Dis. (2017) 40:21–48. 10.1007/s10545-016-9991-4 27905001 PMC5203859

[27] Froese DS Zhang J Healy S Gravel RA. Mechanism of vitamin B12-responsiveness in cblC methylmalonic aciduria with homocystinuria. Mol Genet Metab. (2009) 98:338–43. 10.1016/j.ymgme.2009.07.014 19700356

[28] He R Mo R Shen M Kang L Song J Liu Y. Variable phenotypes and outcomes associated with the MMACHC c609G>A homologous mutation: long term follow-up in a large cohort of cases. Orphanet J Rare Dis. (2020) 15:200. 10.1186/s13023-020-01485-7 32746869 PMC7398195

